# Detecting Biomarkers of Radiation Pneumonitis in Exhaled Breath During Chemoradiotherapy for Stage III Non-small Cell Lung Cancer: Results of a Prospective Feasibility Study

**DOI:** 10.7759/cureus.92938

**Published:** 2025-09-22

**Authors:** Ryan T Hughes, Kaitlyn E Reno, Beverly J Levine, Niema B Razavian, Andrew C Bishop, Jeffrey S Willey, Michael K Farris

**Affiliations:** 1 Radiation Oncology, Wake Forest School of Medicine, Winston Salem, USA; 2 Social Sciences and Health Policy, Wake Forest School of Medicine, Winston Salem, USA; 3 Radiation Oncology, Moffitt Cancer Center, Tampa, USA; 4 Internal Medicine, Section of Molecular Medicine, Wake Forest School of Medicine, Winston Salem, USA

**Keywords:** breath biomarkers, exhaled breath, non-small cell lung cancer, radiation-induced toxicity, radiation pneumonitis, radiotherapy

## Abstract

Introduction

To measure inflammatory biomarkers of radiation pneumonitis in the exhaled breath condensate (EBC) and serum of patients with stage III non-small cell lung cancer (NSCLC) treated with chemoradiotherapy (CRT).

Methods

This single-arm pilot study (NCT04040244) enrolled adults with stage III NSCLC planned for treatment with definitive CRT (60 Gy). EBC and serum samples were collected at baseline (W0) and at 2-, 6-, and 10-weeks after CRT initiation. EBC and serum concentrations of TGF-β1, IL-1α, IL-6, and IL-10, and cartilage oligomeric matrix protein (COMP) were measured in duplicate. Unadjusted mean biomarker levels were described over time.

Results

Samples were available for analysis from 11 out of 16 patients enrolled before the trial was closed early due to the COVID-19 public health emergency. Measurable biomarkers were present in 288 of 302 (95%) available EBC samples and 273 of 278 (98%) serum samples. Mean concentration of TGF-β1, IL-1α, IL-6, IL-10, and COMP over time is described. Mean concentration of TGF-β1 ranged from 81 to 101 pg/mL in EBC and 2819 to 5403 in serum. All biomarkers tested had numerically lower concentrations in EBC than serum, except for IL-10.

Conclusion

Exhaled breath-based analysis of RP-associated biomarkers is feasible, with most available samples having measurable levels of substrate across the multiple biomarkers tested. Given the limited sample size, no tests for association between EBC or serum biomarkers and the development of radiation pneumonitis or pulmonary fibrosis were possible. Larger prospective studies are warranted to determine the association between breath biomarkers and clinical outcomes. Future studies may expand upon this work by integrating clinical and dosimetric factors, using a broader range of inflammatory biomarkers, using a multi-omics approach to identify novel biomarkers, or incorporating breath volatile organic compound profiles.

## Introduction

Definitive chemoradiotherapy (CRT) followed by consolidative immunotherapy is a standard treatment for locally advanced non-small cell lung cancer (NSCLC) [1, 2]. Radiation-induced lung injury (RILI), which includes radiation pneumonitis (RP) and pulmonary fibrosis (PF), occurs with variable severity (ranging from asymptomatic to fatal) in 20% to 50% of patients treated with modern CRT followed by consolidative therapy [3-5]. RP generally refers to a reversible, subacute inflammatory reaction that occurs within several weeks to months of CRT [6]. Symptomatic radiation pneumonitis (SRP), defined as Common Toxicity Criteria for Adverse Events (CTCAE) Grade 2 or higher, occurs in approximately 15-20% of patients and may manifest as cough, shortness of breath, medical therapy or oxygen requirement, or life-threatening respiratory compromise [7, 8]. Current treatment of SRP is largely dependent on symptom severity and can include observation, supportive medications, prolonged corticosteroids, oxygen supplementation, or hospitalization. In addition to RP, immune-mediated pneumonitis occurs in approximately 5% of patients receiving immune checkpoint inhibitor (ICI) therapy after definitive CRT and further compounds the pulmonary risk profile [9]. Additionally, for patients with epidermal growth factor receptor (EGFR)-mutated NSCLC, post-CRT tyrosine kinase inhibitors such as Osimertinib are also associated with risks of pneumonitis [5].

Identifying patients at high risk of SRP prior to treatment is challenging, and data to support individualized prophylactic strategies are limited [10]. The primary method to reduce an individual’s risk of SRP is to reduce radiation exposure to normal lung tissue through the use of complex treatment planning, such as intensity-modulated radiotherapy (IMRT), and to strictly adhere to established normal tissue tolerances [11, 12]. Such constraints were devised prior to the common use of immunotherapy or targeted agents in this patient population. Given that these systemic therapies alone can induce symptomatic pneumonitis, it is unclear if traditional constraints remain appropriate. Development of biomarker-based risk prediction models of SRP in patients treated with modern CRT followed by consolidative systemic therapy may facilitate further investigation of customized prophylactic strategies (i.e., inhaled corticosteroids or short courses of systemic steroids during CRT).

Serum biomarkers, including transforming growth factor-beta 1 (TGF-β1), interleukin (IL)-6, IL-1α, and IL-10, have been associated with the development of SRP [13-16]. Previous studies have combined lung dosimetric parameters and serum TGF-β1 to estimate the risk of SRP [17]. However, studies on these biomarkers were largely conducted prior to the routine use of ICI and targeted agents. This, coupled with substantial inter-study heterogeneity resulting in inconsistency of associations between each individual biomarker and SRP, substantially limited the utility of blood-based risk assessment. Moreover, the inconvenience of blood draws during CRT and the inconsistency of serum biomarkers with SRP in the literature have limited their implementation during routine clinical practice [18-21]. A biosample exhaled directly from the organ of interest may improve upon these blood-based SRP risk assessments and limit the confounding effects of systemic production of inflammatory markers.

Exhaled breath condensate (EBC) is non-invasively captured as the patient exhales over the course of 5-10 minutes into an inexpensive, commercially available device. Prior studies have detected various cytokines, proteins, and other molecules, including TGF-β1, in the EBC of patients with sarcoidosis [22]. Application of this strategy during CRT is an attractive alternative to blood draws and may allow for personalized prophylactic therapies in those at highest risk for SRP. EBC collection could also facilitate the detection of other prognostic biomarkers, such as cartilage oligomeric matrix protein (COMP), which has been correlated with radiation resistance and idiopathic pulmonary fibrosis [18-20, 23].

There is great variability in reported studies assessing for associations between serum biomarkers and SRP, yet there also exists promising evidence that SRP-related biomarkers can be detected in EBC. Since EBC-based biomarker prediction of SRP has not been attempted in stage III NSCLC, a better understanding of the relationship between serum biomarkers, EBC biomarkers, and SRP in this generally high-risk patient population is needed. In this study, we sought to longitudinally measure EBC- and blood-derived biomarkers in patients treated with CRT for stage III NSCLC prior to, during, and after CRT.

## Materials and methods

This prospective pilot study (WFBCCC 98119; NCT04040244) enrolled adults with stage III NSCLC planned for treatment with definitive CRT (total RT dose at least 60 Gy) who were willing and able to provide exhaled breath samples. Patients who were taking systemic corticosteroids within 5 days of registration (to reduce potential confounding with a major endpoint, RP), had prior RT to the chest, or used systemic antibiotics within 2 weeks of registration were excluded. Baseline characteristics, including demographics, tobacco use history, past medical history, and oncologic history, were recorded. Smoking status was ascertained at each collection time point to account for changes in smoking status throughout treatment and follow-up. Results of baseline pulmonary function testing (a part of routine pre-treatment workup) were collected when available. The study was activated in October 2019, with the first patient enrolling in December 2019. The study was subsequently suspended in March 2020 with the outbreak of COVID-19 in the United States. The study was reopened in June 2020 and ultimately closed to accrual in March 2022 due to the ongoing risks of collecting exhaled breath during the COVID-19 public health emergency. This exploratory analysis reports the findings of the patients enrolled prior to study closure.

CRT was performed as per our institutional standard of care, which generally includes 60 Gy in 30 fractions delivered to the thoracic disease with concurrent radiosensitizing weekly carboplatin and paclitaxel. CT simulation was performed with 4-dimensional CT imaging to account for respiratory motion. Radiotherapy was delivered using 3-dimensional conformal RT or intensity-modulated RT (IMRT). Treatment factors (modality, volumetric lung dose parameters, type and schedule of concurrent chemotherapy, use of consolidative immunotherapy) were recorded. Routine clinical assessments and CT imaging were performed 1 month and 4 months after CRT completion. RP was graded using the CTCAE version 5.0, and PF was scored via the Radiation Therapy Oncology Group (RTOG) scale; SRP and symptomatic pulmonary fibrosis (SPF) were both defined as grade 2+ on each respective scale [24, 25]. The primary objective of this exploratory study was to demonstrate the feasibility of detecting RP-associated biomarkers in the EBC of patients treated with CRT by quantifying the intra-person variability of concentrations of TGF-β1, IL-6, IL-1α, IL-10, and COMP. This objective was chosen for two reasons. First, the practical utility of EBC-based biomarker prediction will most likely be the identification of changes from baseline that are associated with a high risk of RP/RF, thus indicating prophylactic treatment prior to the completion of therapy. Second, the lack of strong evidence on EBC biomarkers and their expected variations over time limits our understanding of intra-person variability in this patient population, precluding a well-informed statistical plan and power calculations on an endpoint based on change from baseline over time.

Biospecimen collection occurred at baseline and at 2-, 6-, and 10-weeks after initiation of CRT (W0, W2, W6, and W10, respectively). Whole blood was collected in 8-mL EDTA tubes (BD Vacutainer). EBC was collected using the RTubeTM device (Respiratory Research, Inc., Austin, TX), a single-use apparatus that utilizes a metal cooling sleeve placed over a plastic tube to cool the exhaled breath, condensing and collecting it into a sterile tube. The patient was asked to breathe normally through the mouthpiece for 10 minutes, after which, a metal plunger was used to consolidate the EBC into a fresh sterile tube, which was stored at -80° C until analysis. EBC and serum samples were batched and analyzed after completion of the study. Concentrations of TGF-β1, IL-6, IL-1a, IL-10 and COMP (in pg/mL) were measured in serum and EBC at each time point using enzyme-linked immunosorbent assay (ELISA) kits purchased from bcam (Waltham, MA) for IL-6 (Ab178013), IL-1a (Ab178008), and IL-10 (Ab185986), and R&D Systems (Minneapolis, MN) for TGF-β1 (DB100C) and COMP (DCMP0). Samples were analyzed on a SpectraMax 340PC plate reader (Molecular Devices, San Jose, CA). Measurements were performed in duplicate; standard curves were plotted and used to determine sample concentrations.

Upon early closure of the trial, exploratory analyses were performed on the samples collected as proof of concept for the feasibility of detecting these select biomarkers in EBC. Descriptive statistics were employed for patient characteristics and biomarker levels. Due to the small sample size, no tests comparing biomarker levels in patients who did or did not develop SRP were possible.

## Results

Between October 2019 and March 2022, a total of 16 participants were enrolled. Baseline patient demographics and CRT treatment details are summarized in Table 1. The median age was 63.5 years; 12 were men, 6 were current smokers, and 7 were former smokers. Baseline pulmonary function testing was performed in 11; the median FEV1/FVC ratio was 63.9, and 5 patients had a diagnosis of chronic obstructive pulmonary disease COPD prior to treatment. All patients were planned for treatment with 60 Gy in 30 fractions with concurrent carboplatin/paclitaxel, 11 with IMRT, and 1 with 3D conformal RT. Fourteen of 16 patients completed CRT and received at least one dose of consolidative immunotherapy after completion of CRT.

**Table 1:** Patient and Treatment Characteristics Data are summarized as count (frequency) or mean (standard deviation) unless otherwise specified. COPD: chronic obstructive pulmonary disease; FEV1: forced expiratory volume in one second; FVC: forced vital capacity; NSCLC: non-small cell lung carcinoma; RT: radiotherapy

	Value
Age, median (range)	64 (53-85)
Sex	
Male	12 (75%)
Female	4 (25%)
Race	
Black/African American	3 (23%)
White	13 (77%)
Other	0
Ethnicity	
Not Hispanic or Latino	16 (100%)
Hispanic or Latino	0
Smoking Status at Baseline	
Current smoker	6 (37.5%)
Former smoker	7 (43.8%)
Missing	3 (18.8%)
Pack-years	42.8 (20.1)
Smoking during RT	
All of RT	3 (18.8%)
Some of RT	1 (6%)
Quit before RT	9 (56.3%)
Missing	3 (18.8%)
Histology	
Adenocarcinoma	2 (12.5%)
Squamous cell carcinoma	10 (62.5%)
NSCLC, not otherwise specified	4 (25.0%)
Tumor Classification	
T1	3 (18.8%)
T2	2 (12.5%)
T3	3 (18.8%)
T4	5 (31.3%)
Missing	3 (18.8%)
Nodal Classification	
N0	0
N1	1 (6%)
N2	7 (43.8%)
N3	4 (25%)
Missing	4 (25%)
History of COPD (yes)	5 (31.3%)
Missing	6 (37.5%)
FEV1/FVC	63.9 (11.8)
Radiotherapy Modality	
3DCRT	1 (6%)
IMRT/VMAT	11 (69%)
Missing	4 (25%)
Bilateral Lung Dosimetric Parameters	
V10	44.8 (10.5)
V20	31.0 (3.6)
V30	22.9 (3.5)
Mean lung dose	4.4 (7.6)

Of the 16 patients enrolled, 11 patients provided at least one EBC and at least one serum biospecimen. A total of 302 EBC samples were tested for the various biomarkers, with 288 (95%) having measurable biomarkers present. Similarly, a total of 278 serum samples were tested, and 273 (98%) had measurable biomarkers. The measured concentrations of target biomarkers in EBC and serum at each time point are summarized in Table 2.

**Table 2:** Mean concentration of exhaled breath condensate- and serum-derived biomarkers of symptomatic radiation pneumonitis over time in patients treated with definitive CRT for stage III NSCLC. All measures are presented as mean (standard error (SE)). CRT: chemoradiotherapy; NSCLC: non-small cell lung cancer

	Week 0		Week 2		Week 6		Week 10	
Biomarker	n	Mean (SE)	n	Mean (SE)	n	Mean (SE)	n	Mean (SE)
TGF-B1 Breath	9	80.9 (6.2)	9	81.0 (4.6)	6	86.6 (6.6)	6	100.5 (8.9)
TGF-B1 Serum	9	5403.1 (955.3)	9	4700.1 (1055.3)	7	3007.1 (581.2)	7	2819.1 (332.3)
IL-1a Breath	9	2.3 (0.9)	8	0.9 (0.3)	7	3.0 (2.0)	7	3.6 (2.7)
IL-1a Serum	9	12.5 (6.3)	9	3.4 (0.4)	7	2.7 (0.6)	7	4.2 (1.5)
IL-6 Breath	9	4.8 (0.9)	9	5.3 (0.9)	6	6.5 (0.7)	6	6.4 (0.5)
IL-6 Serum	8	18.0 (5.5)	8	20.1 (4.4)	6	23.7 (12.1)	6	21.1 (5.3)
IL-10 Breath	9	139.9 (7.9)	8	144.5 (5.1)	7	140.3 (5.0)	7	142.4 (13.1)
IL-10 Serum	9	33.3 (1.9)	9	35.5 (2.7)	7	31.7 (1.1)	7	32.1 (1.7)
COMP Breath	9	0.02 (0)	8	0.02 (0)	7	0.03 (0)	7	0.02 (0)
COMP Serum	10	3.7 (0.8)	10	3.4 (0.6)	7	3.2 (0.6)	7	3.9 (0.9)

**Figure 1:**
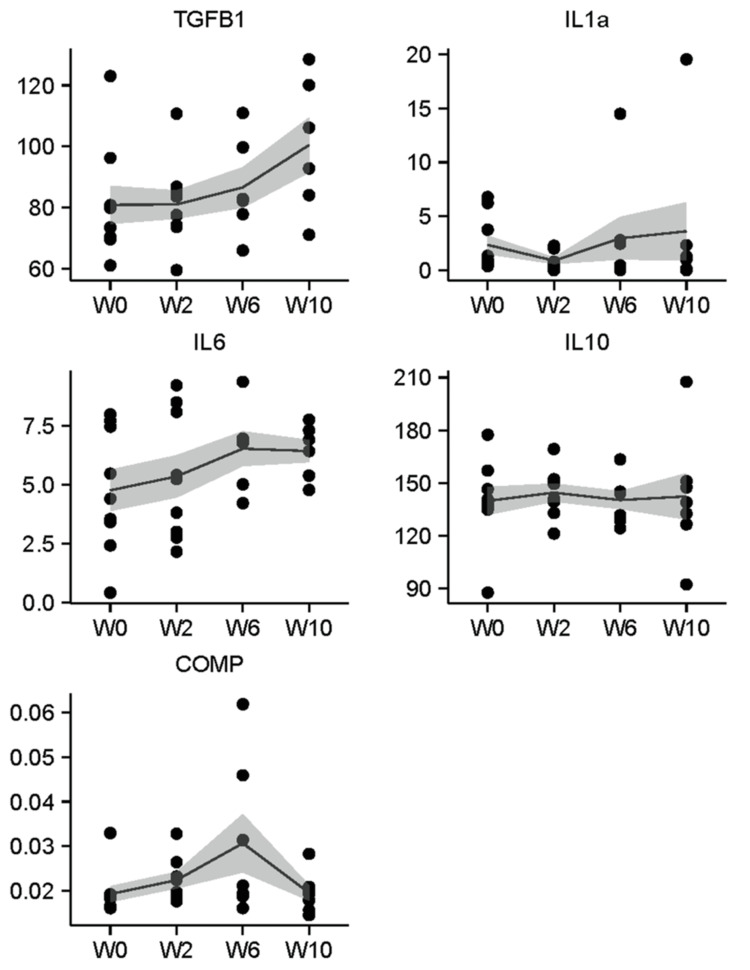
Concentration (pg/mL) of TGF-B1, IL-1a, IL-6, IL-10, and COMP measured in exhaled breath condensate from patients treated with CRT for locally advanced NSCLC. Line indicates mean values over time, shaded ribbon indicates standard error. W0, W2, W6, and W10 indicate the time (in weeks) from initiation of CRT. COMP: cartilage oligomeric matrix protein; CRT: chemotherapy; NSCLC: non-small cell lung cancer

**Figure 2:**
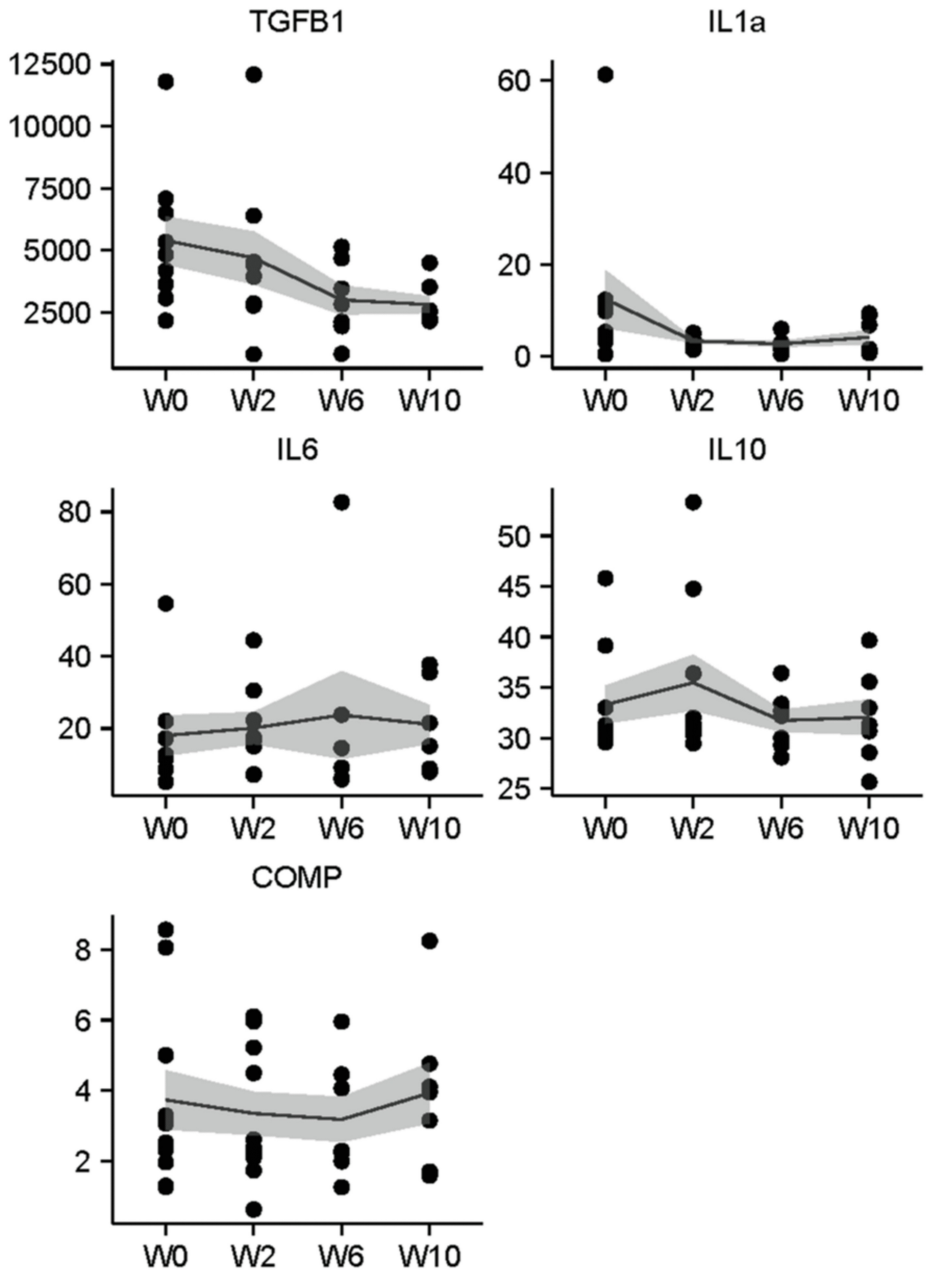
Concentration (pg/mL) of TGF-B1, IL-1a, IL-6, IL-10, and COMP measured in serum from patients treated with CRT for locally advanced NSCLC. Line indicates mean values over time, shaded ribbon indicates standard error. W0, W2, W6, and W10 indicate the time (in weeks) from initiation of CRT. COMP: cartilage oligomeric matrix protein; NSCLC: non-small cell lung cancer

Across all biomarkers tested, the concentration in breath was lower than the concentration in serum, except for IL-10, which at each time point was substantially higher than in serum. Figure 1 depicts individual data points and mean EBC concentration from baseline through W10 for each biomarker. Similarly, Figure 2 depicts data points and mean concentration over time for each biomarker detected in serum.

SRP occurred in three patients (2 with available samples); the highest-grade SRP was two in two patients and three in one patient. Grade 2+ radiation fibrosis occurred in six patients. Of the nine patients with biomarker data at baseline and at least one follow-up (W2 or W6), only one patient developed SRP, and all but one patient developed SPF. Due to this lack of variation in clinical endpoints, no between-group comparisons to test for associations between change from baseline and SRP or SPF were possible.

## Discussion

This study demonstrates that detecting inflammatory biomarkers in the EBC of patients treated with thoracic CRT is feasible and is associated with a high rate of biomarker detection. This study is the first, to our knowledge, to test such a comprehensive battery of RP-associated inflammatory biomarkers in EBC in this patient population.

Given the limited tools currently available to understand the biological mechanisms and predictive biomarkers of RP, there is a need to develop reliable, pragmatic, and biology-driven methods of understanding a patient’s personalized risk. The collection of EBC is a simple, rapid, accessible, and cost-effective (approximately $50-60 per sample) method to collect biological samples that are a rich source of metabolic and genetic information and have the potential to result in novel biomarkers predictive of toxicity outcomes in patients with NSCLC. Although we were unable to directly address whether early changes in EBC biomarkers are associated with the development of RP or fibrosis, this study represents an important step toward advancing the study of breath-based biomarker research.

Various studies have explored EBC-derived biomarker analysis for the detection, diagnosis, or management of lung cancer [26-30]. Others have evaluated EBC analysis as a diagnostic tool in other pulmonary diseases such as idiopathic PF, community-acquired pneumonia, and acute respiratory distress syndrome [31-33]. A prior study examined the use of breath volatile organic compounds (VOCs) to predict RP after stereotactic body radiotherapy (SBRT) for lung tumors [34]. Here, patients who developed SRP had significantly smaller declines in exhaled nitric oxide and carbon monoxide than patients who did not develop SRP. However, collection of VOCs is time-consuming, requires dedicated access to medical air systems and specialized collection and processing equipment, making exhaled VOCs a much less convenient target than proteins detectable in EBC.

An exploratory study by Takahashi et al. performed targeted testing of TGF-β1 levels in the EBC of patients treated with thoracic radiotherapy for lung cancer [35]. They enrolled 10 patients primarily treated with definitive radiotherapy, half with induction or concurrent chemotherapy and half with radiotherapy alone; only 2 patients received consolidative durvalumab. EBC-based TGF-B1 levels increased from a median of 20 pg/mL (range, 0.1-295.1) at baseline to 331.2 pg/mL (range: 33.7-661.3) at 1 month after RT, translating to a median post-RT to pre-RT ratio of 5.9. In a subset analysis, they identified SRP in 5 of 7 patients with an increase in TGF-B1 from baseline to 1-month post-RT; 0 of 2 with a decrease in the same time frame developed SRP. Our study similarly identified non-significant increases in TGF-β1 over time, though not to this amplitude. Our study also reports the novel detection of IL-1a, IL-6, and IL-10, and COMP in EBC of lung cancer patients treated with CRT, expanding the potential portfolio of detectable biomarkers available for study. While both our study and the referenced study were underpowered to detect significant associations between the development of SRP and acute/subacute changes in EBC TGF-B1 levels, their combined data demonstrate feasibility and support the development of larger, prospective studies.

A notable finding from this study pertains to the persistently higher concentration of IL-10 observed in EBC than in serum. IL-10 is an anti-inflammatory cytokine produced by a range of leukocytes that binds to its receptor IL-10R, which is expressed throughout the body (IL-10R1 is expressed predominantly on lymphoid tissues and IL-10R2 systemically), and signals primarily through the JAK1/STAT3 pathway [36,37]. IL-10 and IL-10R have been found to concentrate in proximity to the primary tumor, and IL-10 is produced by alveolar macrophages [38,39]. Higher IL-10 concentrations in EBC than serum are likely related to the short serum half-life of IL-10 and the compartmentalized alveolar environment producing RT-induced IL-10 sampled by EBC [39,40]. IL-10 remained relatively stable in EBC from baseline through 1-month post-treatment follow-up, though this interpretation is limited due to the small sample size. Larger samples will be required to identify a clear association between RT-induced lung inflammation and changes in IL-10. If the hypothesis that a reduction in anti-inflammatory IL-10 signaling is associated with higher SRP risk, this would provide rationale for this pathway as a potential target for novel therapies to reduce the risk of SRP in patients treated with thoracic RT.

This study is not without limitations. Due to the challenges of collecting exhaled breath during a respiratory pandemic, the study sample size is small. Because of this, and the limited number of samples collected per patient, we were likely unable to see variability of SRP/SPF events and could not compare biomarker levels (or their changes from baseline) in groups of patients who did and did not develop RILI. We were also unable to complete post-hoc subgroup analyses based on clinical or dosimetric factors that were collected prospectively. For instance, although smoking status was ascertained at each time point throughout treatment and the follow-up period, the small sample size precluded analyses based on change (or lack thereof) in smoking status throughout the study period on EBC biomarkers. Additionally, the lack of pulmonary function testing for all patients, likely due to patient and pandemic-related factors, limits our ability to interpret our findings in the context of baseline lung function. It is also possible that the EBC and serum batch analyses introduced storage-related variability. Thus, these findings are primarily descriptive and limited to demonstrating the feasibility of the detection of RP-related biomarkers in EBC.

## Conclusions

In conclusion, we identified multiple inflammatory biomarkers in EBC samples taken at multiple time points prior to, during, and after treatment of patients undergoing CRT for stage III NSCLC. Future prospective studies to identify and validate predictive biomarkers of SRP are warranted, which may allow for future studies aimed at expanding our understanding of biomarker-based personalized therapeutic risk assessment and management. Next steps toward this goal may include incorporating clinical risk factors and lung dosimetric parameters, expanding the EBC biomarker panel to additional inflammatory or anti-inflammatory molecules, identification of novel biomarkers through multi-omics analyses, or utilizing breath VOC findings.

## References

[1] Curran WJ Jr, Paulus R, Langer CJ (2011). Sequential vs. concurrent chemoradiation for stage III non-small cell lung cancer: Randomized phase III trial RTOG 9410. J Natl Cancer Inst.

[2] Ramnath N, Dilling TJ, Harris LJ (2013). Treatment of stage III non-small cell lung cancer: Diagnosis and management of lung cancer, 3rd ed: American College of Chest Physicians evidence-based clinical practice guidelines. Chest.

[3] Palma DA, Senan S, Tsujino K (2013). Predicting radiation pneumonitis after chemoradiation therapy for lung cancer: An international individual patient data meta-analysis. Int J Radiat Oncol Biol Phys.

[4] Bradley JD, Paulus R, Komaki R (2015). Standard-dose versus high-dose conformal radiotherapy with concurrent and consolidation carboplatin plus paclitaxel with or without cetuximab for patients with stage IIIA or IIIB non-small-cell lung cancer (RTOG 0617): A randomised, two-by-two factorial phase 3 study. Lancet Oncol.

[5] Lu S, Kato T, Dong X (2024). Osimertinib after chemoradiotherapy in stage III EGFR-mutated NSCLC. N Engl J Med.

[6] Tsoutsou PG, Koukourakis MI (2006). Radiation pneumonitis and fibrosis: Mechanisms underlying its pathogenesis and implications for future research. Int J Radiat Oncol Biol Phys.

[7] Suresh K, Voong KR, Shankar B (2018). Pneumonitis in non-small cell lung cancer patients receiving immune checkpoint immunotherapy: Incidence and risk factors. J Thorac Oncol.

[8] Antonia SJ, Villegas A, Daniel D (2018). Overall survival with durvalumab after chemoradiotherapy in stage III NSCLC. N Engl J Med.

[9] Naidoo J, Wang X, Woo KM (2017). Pneumonitis in patients treated with anti-programmed death-1/programmed death ligand 1 therapy. J Clin Oncol.

[10] Pagel J, Mohorn M, Kloetzer KH, Fleck M, Wendt TG (1998). Inhalative versus oral prophylaxis of pneumonitis in patients receiving thoracic irradiation. Strahlenther Onkol.

[11] Marks LB, Bentzen SM, Deasy JO (2010). Radiation dose-volume effects in the lung. Int J Radiat Oncol Biol Phys.

[12] Chun SG, Hu C, Choy H (2017). Impact of intensity-modulated radiation therapy technique for locally advanced non-small-cell lung cancer: a secondary analysis of the NRG Oncology RTOG 0617 randomized clinical trial. J Clin Oncol.

[13] Anscher MS, Kong FM, Andrews K (1998). Plasma transforming growth factor beta 1 as a predictor of radiation pneumonitis. Int J Radiat Oncol Biol Phys.

[14] Chen Y, Hyrien O, Williams J, Okunieff P, Smudzin T, Rubin P (2005). Interleukin (IL)-1A and IL-6: Applications to the predictive diagnostic testing of radiation pneumonitis. Int J Radiat Oncol Biol Phys.

[15] Hart JP, Broadwater G, Rabbani Z (2005). Cytokine profiling for prediction of symptomatic radiation-induced lung injury. Int J Radiat Oncol Biol Phys.

[16] Arpin D, Perol D, Blay J-Y (2005). Early variations of circulating interleukin-6 and interleukin-10 levels during thoracic radiotherapy are predictive for radiation pneumonitis. J Clin Oncol.

[17] Fu XL, Huang H, Bentel G (2001). Predicting the risk of symptomatic radiation-induced lung injury using both the physical and biologic parameters V-30 and transforming growth factor beta. Int J Radiat Oncol Biol Phys.

[18] Hanitrarimalala V, Bednarska I, Murakami T, Papadakos KS, Blom AM (2024). Intracellular cartilage oligomeric matrix protein augments breast cancer resistance to chemotherapy. Cell Death Dis.

[19] Li Q, Wang C, Wang Y (2018). HSCs-derived COMP drives hepatocellular carcinoma progression by activating MEK/ERK and PI3K/AKT signaling pathways. J Exp Clin Cancer Res.

[20] Cui J, Zhang J (2022). Cartilage oligomeric matrix protein, diseases, and therapeutic opportunities. Int J Mol Sci.

[21] Zheng C, Li X, Ren Y, Yin Z, Zhou B (2020). Coexisting EGFR and TP53 mutations in lung adenocarcinoma patients are associated with COMP and ITGB8 upregulation and poor prognosis. Front Mol Biosci.

[22] Ahmadzai H, Cameron B, Chui J, Lloyd A, Wakefield D, Thomas PS (2013). Measurement of neopterin, TGF-β1 and ACE in the exhaled breath condensate of patients with sarcoidosis. J Breath Res.

[23] Vuga LJ, Milosevic J, Pandit K (2013). Cartilage oligomeric matrix protein in idiopathic pulmonary fibrosis. PLoS One.

[24] (2025). National Cancer Institute, Division of Cancer Treatment & Diagnosis, U.S. Department of Health and Human Services: Common terminology criteria for adverse events (CTCAE) Version 5.0. https://ctep.cancer.gov/protocoldevelopment/electronic_applications/ctc.htm#ctc_50.

[25] Cox JD, Stetz J, Pajak TF (1995). Toxicity criteria of the Radiation Therapy Oncology Group (RTOG) and the European Organization for Research and Treatment of Cancer (EORTC). Int J Radiat Oncol Biol Phys.

[26] Dalaveris E, Kerenidi T, Katsabeki-Katsafli A, Kiropoulos T, Tanou K, Gourgoulianis KI, Kostikas K (2009). VEGF, TNF-alpha and 8-isoprostane levels in exhaled breath condensate and serum of patients with lung cancer. Lung Cancer.

[27] Zhang D, Takigawa N, Ochi N (2011). Detection of the EGFR mutation in exhaled breath condensate from a heavy smoker with squamous cell carcinoma of the lung. Lung Cancer.

[28] Kordiak J, Szemraj J, Grabska-Kobylecka I, Bialasiewicz P, Braun M, Kordek R, Nowak D (2019). Intratumor heterogeneity and tissue distribution of KRAS mutation in non-small cell lung cancer: Implications for detection of mutated KRAS oncogene in exhaled breath condensate. J Cancer Res Clin Oncol.

[29] Youssef O, Knuuttila A, Piirilä P, Böhling T, Sarhadi V, Knuutila S (2017). Presence of cancer-associated mutations in exhaled breath condensates of healthy individuals by next generation sequencing. Oncotarget.

[30] Smyth RJ, Toomey SM, Sartori A (2018). Brief report on the detection of the EGFR T790M mutation in exhaled breath condensate from lung cancer patients. J Thorac Oncol.

[31] Rindlisbacher B, Strebel C, Guler S (2017). Exhaled breath condensate as a potential biomarker tool for idiopathic pulmonary fibrosis—a pilot study. J Breath Res.

[32] Aliberti S, Morlacchi LC, Faverio P (2016). Serum and exhaled breath condensate inflammatory cytokines in community-acquired pneumonia: A prospective cohort study. Pneumonia (Nathan).

[33] Bos LD (2018). Diagnosis of acute respiratory distress syndrome by exhaled breath analysis. Ann Transl Med.

[34] Moré JM, Eclov NC, Chung MP (2014). Feasibility and potential utility of multicomponent exhaled breath analysis for predicting development of radiation pneumonitis after stereotactic ablative radiotherapy. J Thorac Oncol.

[35] Takahashi S, Anada M, Kinoshita T, Nishide T, Shibata T (2022). Prospective exploratory study of the relationship between radiation pneumonitis and TGF-β1 in exhaled breath condensate. In Vivo.

[36] Hutchins AP, Diez D, Miranda-Saavedra D (2013). The IL-10/STAT3-mediated anti-inflammatory response: recent developments and future challenges. Brief Funct Genomics.

[37] Murray PJ (2006). Understanding and exploiting the endogenous interleukin-10/STAT3-mediated anti-inflammatory response. Curr Opin Pharmacol.

[38] Vahl JM, Friedrich J, Mittler S (2017). Interleukin-10-regulated tumour tolerance in non-small cell lung cancer. Br J Cancer.

[39] Yanagawa H, Takeuchi E, Suzuki Y, Hanibuchi M, Haku T, Qhmoto Y, Sone S (1999). Production of interleukin-10 by alveolar macrophages from lung cancer patients. Respir Med.

[40] Huhn RD, Radwanski E, O'Connell SM (1996). Pharmacokinetics and immunomodulatory properties of intravenously administered recombinant human interleukin-10 in healthy volunteers. Blood.

